# Nilotinib Counteracts P-Glycoprotein-Mediated Multidrug Resistance and Synergizes the Antitumoral Effect of Doxorubicin in Soft Tissue Sarcomas

**DOI:** 10.1371/journal.pone.0037735

**Published:** 2012-05-25

**Authors:** Victor Hugo Villar, Oliver Vögler, Jordi Martínez-Serra, Rafael Ramos, Silvia Calabuig-Fariñas, Antonio Gutiérrez, Francisca Barceló, Javier Martín-Broto, Regina Alemany

**Affiliations:** 1 Clinical and Translational Research Group, Department of Biology, Institut Universitari d'Investigacions en Ciències de la Salut (IUNICS), University of the Balearic Islands, Palma de Mallorca, Spain; 2 Department of Hematology, University Hospital Son Espases, Palma de Mallorca, Spain; 3 Department of Pathology, University Hospital Son Espases, Palma de Mallorca, Spain; 4 Department of Oncology University Hospital Son Espases, Palma de Mallorca, Spain; Vanderbilt University Medical Center, United States of America

## Abstract

The therapeutic effect of doxorubicin (DXR) in the treatment of soft tissue sarcomas (STS) is limited by its toxicity and the development of multidrug resistance (MDR), the latter mainly induced by high expression of efflux pumps (e.g., P-glycoprotein [P-gp]). Therefore, the search for alternative therapies, which sensitize these tumors to chemotherapy while maintaining a low toxicity profile, is a rational approach. We assessed efficacy and molecular mechanisms involved in the antiproliferative effects of the tyrosine kinase inhibitors, nilotinib and imatinib, as single agents or in combination with DXR, in human synovial sarcoma SW982 and leiomyosarcoma SK-UT-1 cells. As single compound nilotinib (1–10 µM) was more potent than imatinib inhibiting the growth of SK-UT-1 and SW982 cells by 33.5–59.6%, respectively. Importantly, only nilotinib synergized the antitumoral effect of DXR (0.05–0.5 µM) by at least 2-fold, which clearly surpassed the mere sum of effects according to isobolographic analysis. Moreover, nilotinib in combination with DXR had a sustained effect on cell number (−70.3±5.8%) even 12 days after withdrawal of drugs compared to DXR alone. On the molecular level, only nilotinib fully blocked FBS-induced ERK1 and p38 MAPK activation, hence, reducing basal and DXR-induced up-regulation of P-gp levels. Moreover, efflux activity of the MDR-related proteins P-gp and MRP-1 was inhibited, altogether resulting in intracellular DXR retention. In high-risk STS tumors 53.8% and 15.4% were positive for P-gp and MRP-1 expression, respectively, with high incidence of P-gp in synovial sarcoma (72.7%). In summary, nilotinib exhibits antiproliferative effects on cellular models of STS and sensitizes them to DXR by reverting DXR-induced P-gp-mediated MDR and inhibiting MRP-1 activity, leading to a synergistic effect with potential for clinical treatment.

## Introduction

Sarcomas are a heterogeneous group of malignant mesenchymal tumors. Within this group, soft tissue sarcomas (STS) are cancers of muscle, fat, fibrous or other supporting tissues of the body. Although the most common treatment is surgical removal of the entire tumor, doxorubicin (DXR)-based chemotherapy has been the current treatment for patients with locally advanced inoperable or metastatic disease [1]. However, the clinical effectiveness of DXR is limited by severe toxicity and the development of multidrug resistance (MDR), the latter mainly involving high cellular expression of ATP-binding cassette (ABC) transporters in the plasma membrane, including P-glycoprotein (P-gp) and multidrug resistance-related protein 1 (MRP-1) [2], [3]. These proteins are ATP-dependent pumps that carry xenobiotic agents, such as the antineoplastic compound DXR, out of the cells, thereby reducing its antitumoral effect. Accordingly, the search for combination therapies, which are able to counteract such resistance mechanism in cancer cells without increasing general toxicity, is a rational clinical approach.

Anticancer therapy based on molecular targeting comprises selective inhibition of specific tyrosine kinases (TKs), which play a crucial role in tumor growth or progression [4]. Therefore, TK inhibitors have become a promising therapeutic option for treatment of cancer types whose molecular pathogenesis implicates the overexpression or activation of various TKs (e.g., BCR/ABL) or TK receptors (e.g., c-KIT, PDGFR and EGFR, among others) [5]. Usually, inhibition of oncogenic TK activity leads to down-regulation of several downstream signaling pathways, including mitogen-activated protein kinase (MAPK) cascades and phosphatidylinositol 3′-kinase (PI3K)/AKT pathway, consequently repressing proliferation, invasion and survival of cancer cells. Accordingly, the TK inhibitor imatinib mesylate (STI571; Gleevec; Novartis) has become first-line therapy for patients with chronic myeloid leukaemia (CML) harbouring BCR/ABL translocation [6] or for those with advanced gastrointestinal stromal tumor (GIST) showing specific mutations in c-KIT or PDGFRα genes, which activate these TKs [7]. Despite the fact that imatinib initially improves dramatically the outcome of these patients, its beneficial effect is limited by intrinsic and acquired drug resistance, which prevails in most of the patients and finally leads to relapse or interruption of treatment [8], [9]. These findings promoted the development of a second generation of TK inhibitors, such as sunitinib (SU11248, Sutent; Pfizer) [10] and nilotinib (AMN107, Tasigna, Novartis) [11]. Nilotinib has been reported to inhibit BCR/ABL kinase more potently than imatinib being at least similarly effective concerning c-KIT and PDGFR kinases [12]. Nilotinib differs from imatinib regarding its cellular transport, leading to higher intracellular levels (5 to 10-fold) of this agent [13]. In parallel, nilotinib still exhibited antitumoral efficacy in patients with CML [14] and GIST, who were resistant to imatinib or sunitinib [15]. Very recently it has been demonstrated that nilotinib has also potential to reverse MDR by inhibiting the activity of P-gp and ABCG2 transporters in human embryonic kidney (HEK) 293 cells that exogenously overexpress these efflux pumps [16].

**Figure 1:**
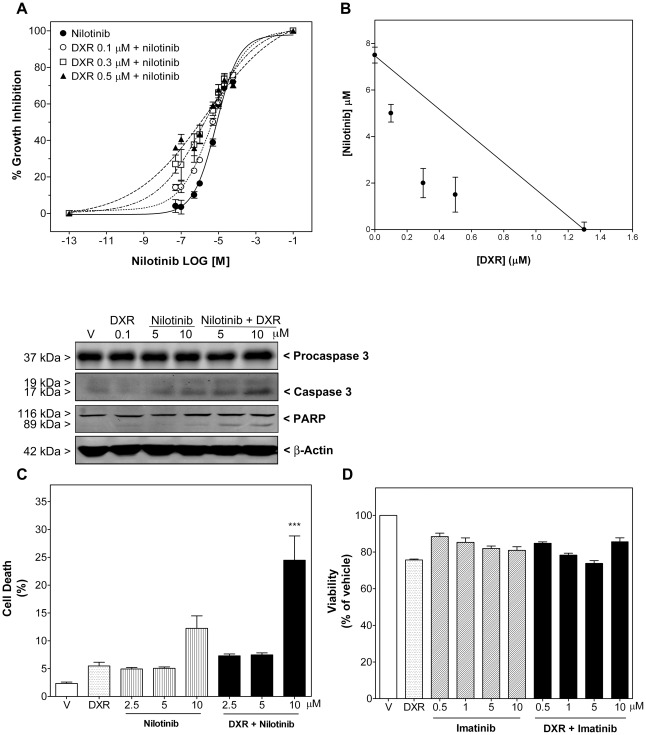
Combination of nilotinib, but not imatinib, with doxorubicin (DXR) displayed a synergistic effect on growth inhibition and apoptosis in synovial sarcoma SW982 cells. A) Antiproliferative effect of nilotinib as single compound or combined with DXR. Synovial sarcoma cells were treated with vehicle or nilotinib (0.1–40 µM) alone or combined simultaneously with DXR (0.1, 0.3 and 0.5 µM) for 72 h. Each value represents mean ± SEM of four individual experiments performed in triplicate. B) Subsequent isobolographic analyses were performed as described in the Material and Methods section. Drug combination was synergistic at all concentrations (CI<1), as shown in the isobologram. C) Apoptotic effect of nilotinib as single compound or combined with DXR (0.1 µM) for 72 h. *Upper panels* show the immunoreactive bands of procaspase 3, cleaved caspase 3 and PARP fragmentation in representative immunoblots. DNA content of cells was measured by flow cytometry. Columns show percentage of apoptotic cells in the absence (vehicle, V) or presence of DXR and nilotinib as single compounds or combined (DXR+nilotinib). Each column represents mean ± SEM of four independent experiments. ^***^ *P*<0.001 versus DXR-treated cells. D) Antiproliferative effect of imatinib (0.5–10 µM) as single compound or combined with DXR (0.1 µM) for 72 h. Each column represents mean ± SEM of three independent experiments.

**Figure 2:**
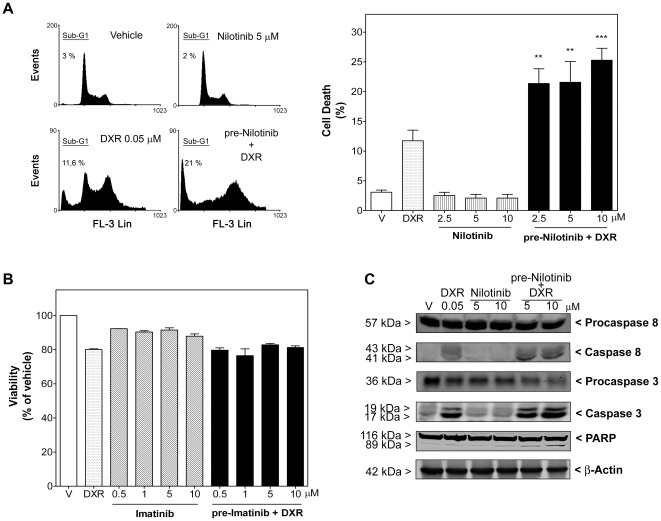
Combination of nilotinib, but not imatinib, with doxorubicin displayed a synergistic effect on apoptosis in leiomyosarcoma SK-UT-1 cells. A, *left*, DNA content of cells was measured by flow cytometry. Representative histograms of vehicle-treated (DMSO), DXR (0.05 µM)-treated, nilotinib (5 µM)-treated and pre-nilotinib 24 h+DXR-treated cells are shown. The fluorescence values used to calculate the peak corresponding to the sub-G1 phase are indicated on each histogram. *Right*, Columns show percentage of apoptotic cells in the absence (vehicle, V) or presence of DXR and nilotinib as single agents or combined (pre-Nilotinib+DXR). Each column represents mean ± SEM of four independent experiments. ^**^ *P*<0.01 and ^***^ *P*<0.001 versus DXR-treated cells. B) Antiproliferative effect of imatinib as single compound or combined with DXR (0.05 µM) (pre-Imatinib+DXR) for 72 h. Each column represents mean ± SEM of three independent experiments. C) Panels show the immunoreactive bands of procaspase 8 and 3, cleaved caspase 8 and 3, and PARP fragmentation in representative immunoblots of leiomyosarcoma cells treated as in A.

**Figure 3:**
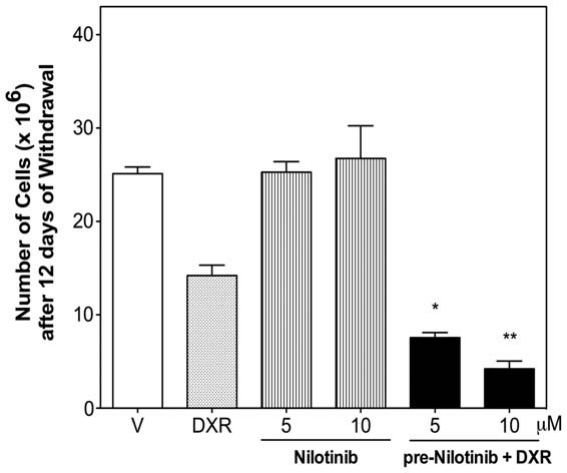
Nilotinib plus doxorubicin-treated leiomyosarcoma SK-UT-1 cells showed prolonged inhibition of cell growth after drug withdrawal. Cells were pre-treated with vehicle or nilotinib for 24 h, and DXR (0.05 µM) was then added to the corresponding wells and treatment continued for another 72 h. Cells were then recovered, diluted and reseeded at a density of 1.8×10^4^ cell per well with culture medium without any further compounds. After 12 days, cells were counted using a hemocytometer. Values are mean ± SEM of three independent experiments. ^*^ *P*<0.05 and ^**^ *P*<0.01 versus DXR-treated cells.

**Figure 4:**
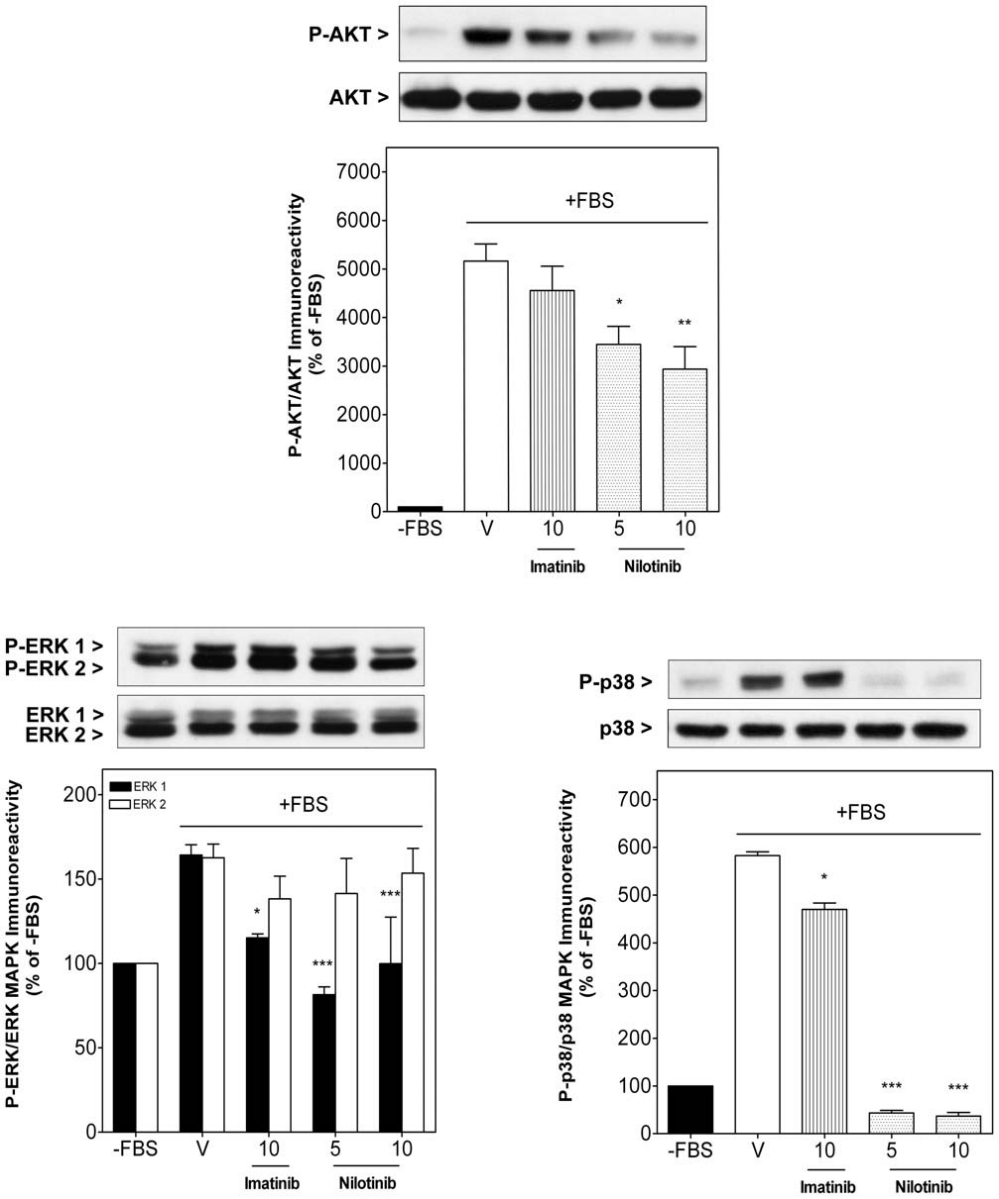
Nilotinib decreased fetal bovine serum-induced AKT activation and fully blocked ERK1/2 and p38 MAPK activation. Sub-confluent synovial sarcoma SW982 cells were deprived of fetal bovine serum (FBS) for 4 h. Cells were then stimulated for 30 min with 10% FBS in the absence (vehicle, V) or presence of imatinib (10 µM) or nilotinib (5 and 10 µM) as single compounds. *Upper panels* show representative immunoblots of four independent experiments. Columns represent the phosphorylated ratio of AKT, ERK1/2 and p38 MAPK. Each column represents mean ± SEM of four independent experiments normalized to FBS-depleted cells (-FBS, taken as 100%). ^*^ *P*<0.05; ^**^ *P*<0.01 and ^***^ *P*<0.001 versus vehicle-treated cells stimulated with FBS.

**Figure 5:**
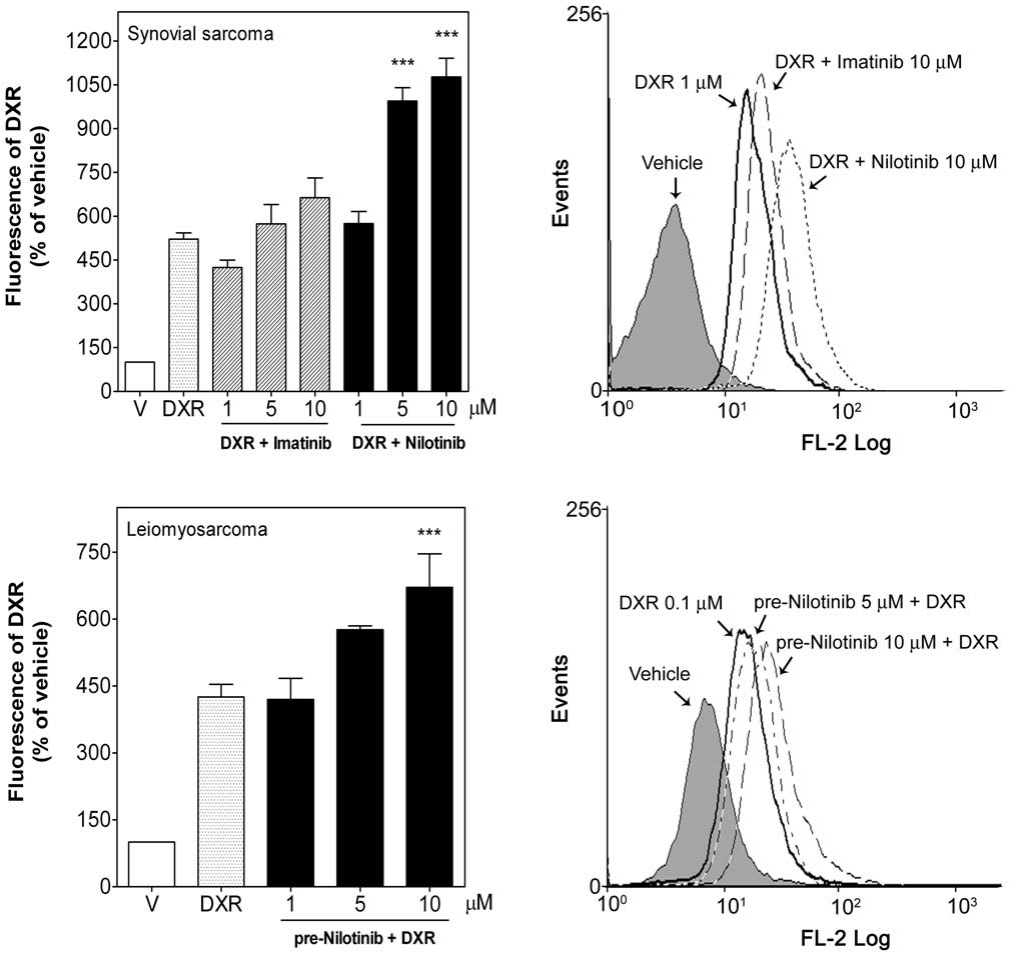
Nilotinib, but not imatinib, increased intracellular doxorubicin in human STS cells. Synovial sarcoma SW982 and leiomyosarcoma SK-UT-1 cells were incubated with vehicle (DMSO) or DXR (1 µM) in the absence (vehicle, V) or presence of nilotinib (1–10 µM) or imatinib (1–10 µM) as described in the Material and Methods section. After 24 h incubation, intracellular DXR was measured by its fluorescence intensity with flow cytometry. *Left*, Median of intracellular DXR fluorescence in the absence (V) or presence of TK inhibitors normalized to vehicle-treated cells (taken as 100%). Each column represents mean ± SEM of seven independent experiments. *Right*, Representative flow cytometry analysis of the intracellular DXR fluorescence detected with excitation at 488 nm and emission at 580 nm in both cell lines. ^***^ *P*<0.001 versus DXR-treated cells.

**Figure 6:**
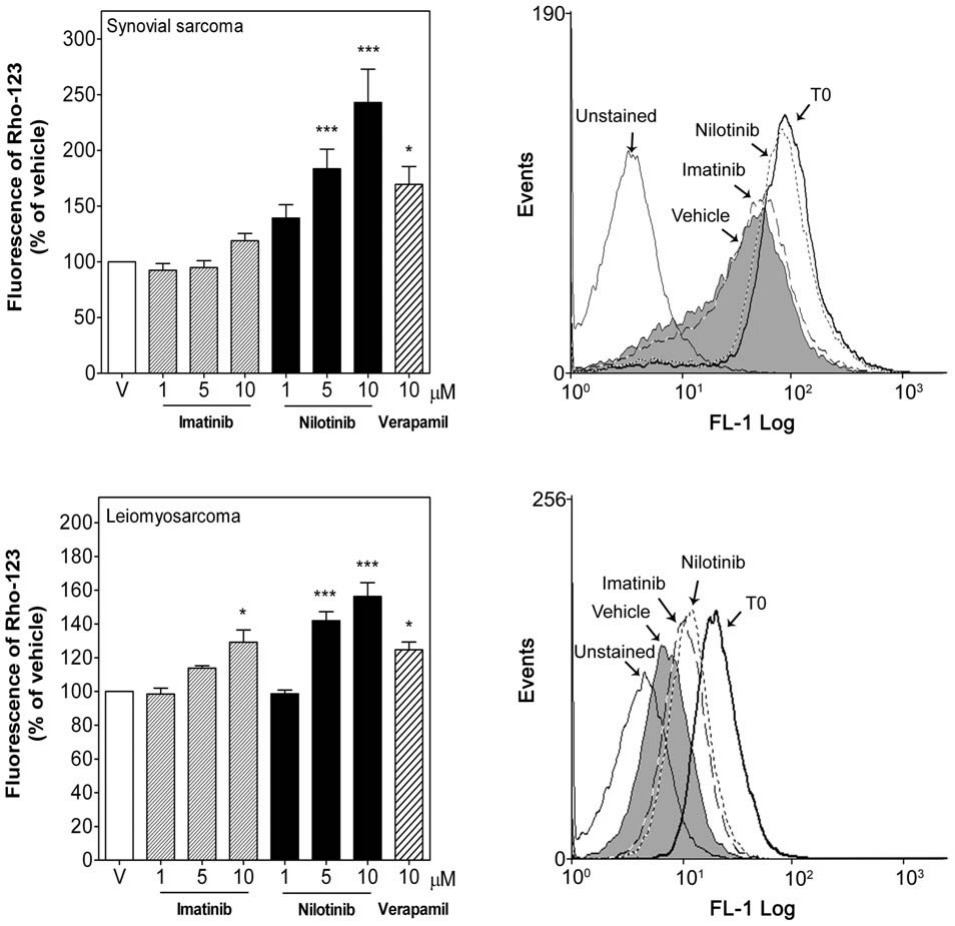
Nilotinib inhibited P-gp activity in human STS cells. Intracellular Rho-123 fluorescence (0.5 or 0.05 µM) was estimated by flow cytometry in synovial sarcoma SW982 cells after 2 h treatment or in leiomyosarcoma SK-UT-1 cells after 24 h treatment with vehicle (DMSO), imatinib (1–10 µM), nilotinib (1–10 µM) or verapamil (10 µM). *Left*, Median of intracellular Rho-123 fluorescence in the absence (vehicle, V) or presence of TK inhibitors normalized to vehicle-treated cells (taken as 100%). Each column represents mean ± SEM of five independent experiments. *Right*, Representative flow cytometry analysis of the intracellular Rho-123 fluorescence in the absence (vehicle) or presence of imatinib (10 µM) or nilotinib (10 µM) detected with excitation at 488 nm and emission at 580 nm. T0 was the intracellular Rho-123 fluorescence measured at t = 0. ^*^ *P*<0.05 and ^***^ *P*<0.001 versus vehicle-treated cells.

**Figure 7:**
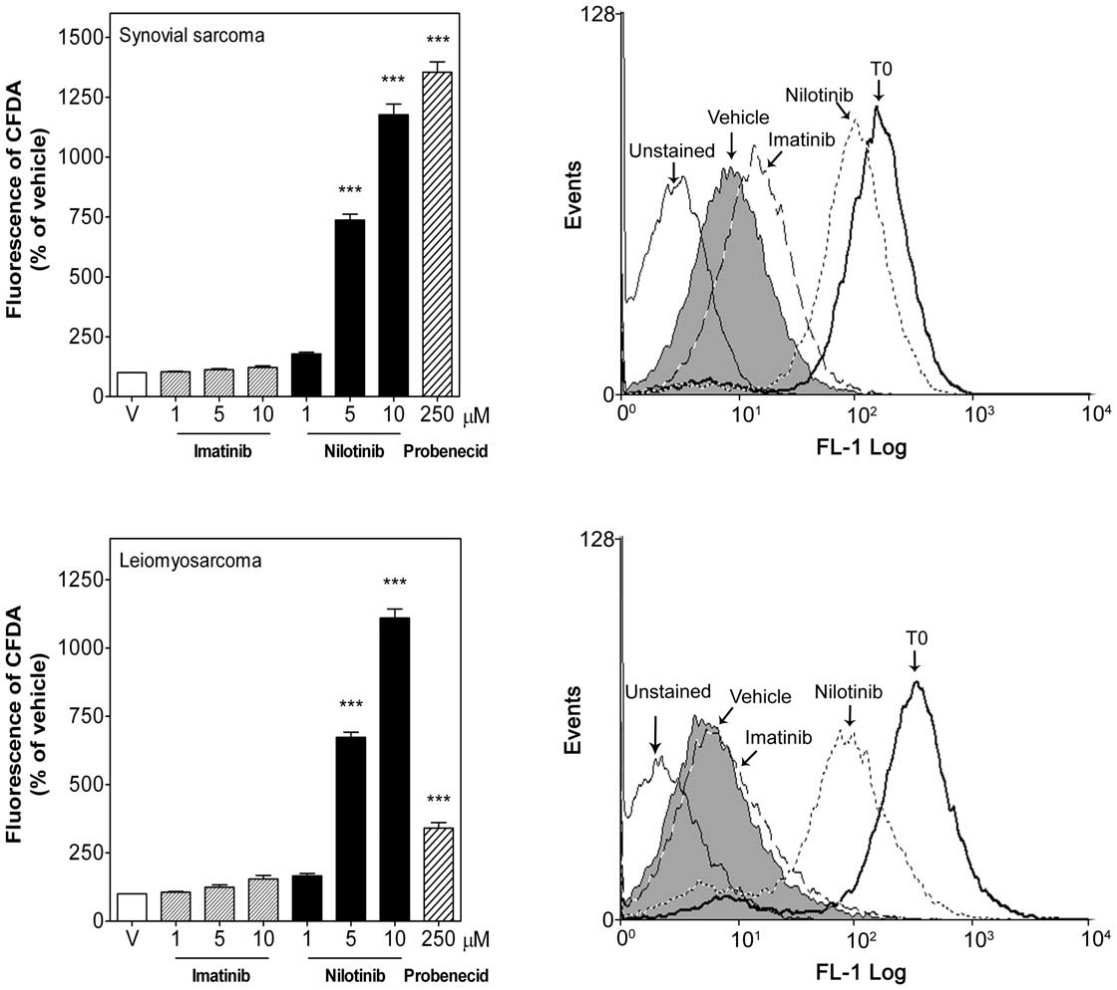
Nilotinib inhibited MRP-1 activity in human STS cells. Intracellular 5(6)-CFDA fluorescence was estimated by flow cytometry in synovial sarcoma SW982 and leiomyosarcoma SK-UT-1 cells after 2 h treatment with vehicle (V), imatinib (1–10 µM), nilotinib (1–10 µM) or probenecid (250 µM). *Left*, Median of intracellular CFDA fluorescence in the absence or presence of TK inhibitors normalized to vehicle-treated cells (taken as 100%). Each column represents mean ± SEM of three independent experiments. *Right*, Representative flow cytometry analysis of the intracellular CFDA fluorescence in the absence (vehicle) or presence of imatinib (10 µM) or nilotinib (10 µM) detected with excitation at 488 nm and emission at 530 nm. T0 was the intracellular CFDA fluorescence measured at t = 0. ^***^ *P*<0.001 versus vehicle-treated cells.

**Figure 8:**
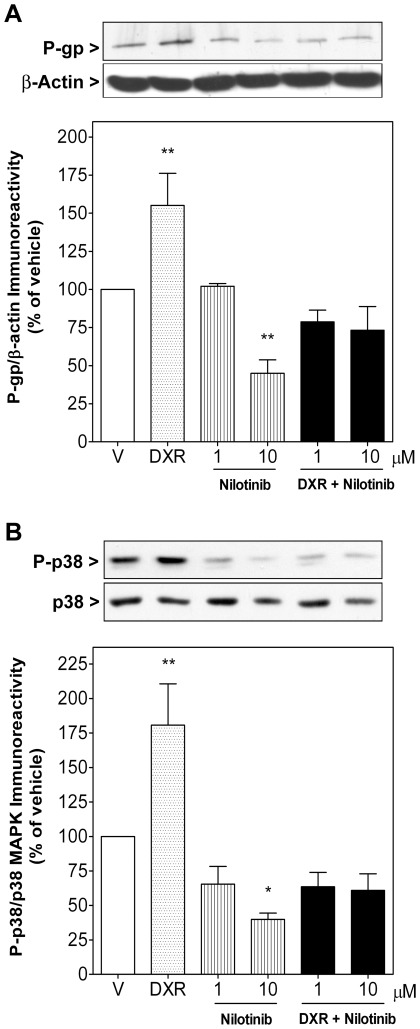
Nilotinib down-regulated basal P-gp levels and p38 MAPK phosphorylation and fully blocked both DXR-induced up-regulation of P-gp levels and p38 MAPK phosphorylation. Sub-confluent synovial sarcoma SW982 cells were treated for 72 h with vehicle (V), DXR (0.1 µM), nilotinib (10 µM) as single compounds or combined simultaneously (DXR+Nilotinib). *Upper panels* show representative immunoblots of four independent experiments. A) Columns represent the ratio of the P-gp to β-actin immunoreactivity or B) the phosphorylation ratio of p38 MAPK. Each column represents mean ± SEM of four independent experiments normalized to vehicle-treated cells (taken as 100%). ^*^ *P*<0.05 and ^**^ *P*<0.01 versus vehicle-treated cells.

**Table 1:** Detection of P-gp and MRP-1 in high-risk locally advanced soft tissue sarcoma tumors.

Histological type	N° of cases	P-gp +	MRP-1 +	Coexpression
Synovial sarcoma	11	8	3	3
Pleomorphic rabdomyosarcoma	2	0	1	0
Leiomyosarcoma	4	4	1	1
Pleomorphic liposarcoma	5	3	0	0
Malignant fibrous histiocytoma	19	7	2	2
Fibrosarcoma	2	0	0	0
Non-classified sarcomas	9	6	1	0
Total cases	52	28	8	6

P-gp and MRP-1 expression was analyzed by immunostaining in a subset of fifty two high-risk localized STS tumors of limbs or trunk wall, which derived from a total of three hundred and twenty-eight patients enrolled in a randomized international trial, as described in the Material and Methods section. P-gp and MRP-1 expression was evaluated qualitatively: positive or negative.

Although some studies have evaluated the effectiveness of imatinib and sunitinib in STS other than GIST [17], [18] only little is known regarding the effectiveness of nilotinib and whether a combination of TK inhibitors with conventional chemotherapy may improve treatment outcome for this type of solid tumors. Our study compares the effectiveness and molecular mechanisms involved in the antiproliferative effects of the TK inhibitors, nilotinib and imatinib, as individual therapeutic agents or in combination with DXR, in human cell lines of STS, being susceptible to development of drug resistance.

## Materials and Methods

### Cell culture and treatments

The human synovial sarcoma SW982 and leiomyosarcoma SK-UT-1 cell lines were obtained from the American Type Culture Collection (Manassas, VA). Synovial sarcoma cells were grown in Leibovitz's L-15 medium (Invitrogen S.A, Barcelona, Spain), whereas leiomyosarcoma cells were cultured in DMEM medium (LabClinics S.A, Barcelona, Spain) supplemented with MEM-non essential aminoacids (dilution 1∶100) and 1 mM sodium pyruvate. Both media contained 2 mM L-glutamine and were supplemented with 10% (v/v) fetal bovine serum, 100 units/ml penicillin and 100 µg/ml streptomycin. Tissue culture supplements were all purchased from Sigma-Aldrich (Madrid, Spain). When the cells reached 60–70% confluence, vehicle (DMSO), nilotinib (NVP-AMN107-AA), imatinib mesylate or doxorubicin (DXR) as single agents or their combinations were added to the medium for 24–96 h. Nilotinib and imatinib were kindly provided by Novartis Pharma AG (Basel, Switzerland). DXR was purchased from Sigma-Aldrich. Stock solutions of nilotinib and imatinib were prepared at 10 mM in dimethylsulfoxide (DMSO) or in water in the case of DXR.

### Cell viability

Synovial sarcoma and leiomyosarcoma cells were plated in 96-well plates at a density of 11×10^3^ or 8×10^3^ cells per well in 200 µl of their respective growth medium, and grown for 24 h. To test the effectiveness of DXR and TK inhibitors on cell growth, cells were exposed to increasing concentrations of DXR, nilotinib or imatinib (0.05–90 µM) for 72 h. The compound concentration resulting in 50% inhibition of cell viability (IC_50_) was determined using GraphPad software. To determine whether nilotinib and imatinib would increase sensitivity of these cell lines to DXR, cells were treated with varying concentrations of TK inhibitors (range 0.1–40 µM) with DXR (0.05–0.5 µM) or without it for 72 h. Synovial sarcoma cells were treated simultaneously with DXR and TK inhibitors, whereas leiomyosarcoma cells were pre-treated with vehicle or TK inhibitor for 24 h, and DXR was then added to the corresponding wells and treatment continued for another 48 h. These experimental conditions appeared to be the most effective combination in a previous study. After 72 h, the viability of the cells was measured using the methylthiazoletetrazolium (MTT) method. Absorbance was measured at 570 (for SW982 cells) and 590/650 nm (for SK-UT-1 cells) on an ELISA plate reader (Asys Hitech GmbH, Austria). The mean percentage of cell survival relative to that of vehicle-treated cells was estimated from data of three individual experiments performed by triplicate.

### Isobolographic analysis

The effects of the combination of nilotinib with DXR in synovial sarcoma cell growth were analyzed by Loewe's isobolographic analysis revised by Steel and Peckman [19], which distinguishes three types of interactions: pure additivity, synergy and antagonism. In this model, the combination index (CI) is described by CI = dA/DA+dB/DB, where dA and dB are the concentration of drug A and B given in a combination of two drugs and DA and DB are the concentration of drug A and B yielding the same effect level, when administered alone, as the mixture. Combinations with CI>1 are considered as antagonistic, those with CI = 1 are additive, and those with CI<1 are synergistic. If synergy exists, then a lower concentration of dA and/or dB would be required to achieve the same effects of the theoretical dosages for additivity [20]. In our studies CI values for each condition were calculated using the IC_50_ of cell growth as the isoeffective point and these values were determined by plotting the results obtained in the MTT assay in a Hill Curve. The isobolograms were constructed by plotting the IC_50_ of nilotinib on the Y-axis and the IC_50_ of DXR on the X-axis, being the line that connects these two points the line of additivity.

### Cell proliferation assays

To study the effect of the withdrawal of nilotinib, DXR or nilotinib plus DXR on cell proliferation, leiomyosarcoma cells were pre-treated with vehicle or nilotinib for 24 h, and DXR was then added to the corresponding wells and treatment continued for another 72 h. After treatment, cells were recovered, diluted and reseeded at a density of 1.8×10^4^ cell/well with culture medium without any further compounds. After 12 days of cell culture, cells were counted using a hemocytometer.

### Cell cycle analysis and apoptosis

The apoptotic index and analysis of the cell cycle were performed on STS cells by flow cytometry as described previously [21]. Cell populations in the different phases of cell cycle (sub-G1 (cell death), G0/G1, S/G2/M peaks) were determined based on their DNA content in a Beckman Coulter Epics XL flow cytometer. Cellular apoptosis was also determined by assessment of the cleavage of caspase 3, caspase 8 and PARP by immunoblot analysis and measurement of caspases 3 and 7 activities by means of the luminometric Caspase-Glo 3/7 assay (Promega, Barcelon, Spain) according to the manufacturer's instructions, using a Synergy HT multidetection microplate reader (Bio-Tek, Winooski, VT, USA).

### Immunoblot analysis

Preparation of cell extracts and protein assays were performed as described previously [21] with the following change: phosphatase inhibitor cocktails 1 and 2 (Sigma, Madrid) were added to the ice-cold lysis buffer (dilution 1∶100). Primary polyclonal antibodies anti-poly ADP-ribose polymerase (anti-PARP) (116 and 89 KDa), anti-caspase 3 (35 kDa), anti-cleaved caspase-3 (Asp175) (17–19 kDa), anti-phospho-AKT (P-AKT) (Ser473; 60 kDa), anti-AKT (60 kDa), anti-phospho p38 MAPK (P-p38 MAPK) (Thr180/Tyr182; 38 kDa), anti-p38 MAPK (p38 MAPK) (38 kDa), anti-phospho-p44/42 MAPK (P-ERK1/2) (Thr202/Tyr204), anti-p44/42 MAPK (ERK1/2) and the monoclonal antibodies anti-caspase 8 (full lenght 57 kDa; the cleaved intermediate 43 and 41 kDa and the active fragment 18 kDa) and anti-β-actin (42 kDa) were obtained from Cell Signaling Technology (Danvers, MA). The monoclonal anti-MDR1 (P-gp) (170 kDa) was purchased from Santa Cruz Biotechnology, Inc. (Santa Cruz, CA). Antibodies were all diluted 1∶1000. The intensity of band immunoreactivity was analyzed by densitometry with TotalLab software from Nonlinear dynamics (Amersham). To assess the changes of phosphorylation or protein levels, the intensity of each phosphorylated protein or protein band was normalized against the intensity of the corresponding total protein or β-actin band, respectively. This procedure was repeated at least three times for each sample on different gels.

### Quantification of intracellular doxorubicin

Synovial sarcoma cells were treated with vehicle (DMSO) or DXR (1 µM) in the absence or presence of nilotinib (1–10 µM) and imatinib (1–10 µM) for 24 h. Leiomyosarcoma cells were pre-treated with vehicle or nilotinib for 24 h, and DXR (0.1 µM) was then added to the corresponding wells and treatment continued for another 24 h. After this time, cells were harvested, washed and resuspended in PBS at 37°C. Fluorescence attributable to intracellular DXR was immediately measured by flow cytometry (Beckman Coulter Epics XL flow cytometer).

### Activity of P-glycoprotein and multidrug resistance-associated protein 1

To examine the effect of TK inhibitors on P-glycoprotein (P-gp) and multidrug resistance-associated protein 1 (MRP-1) function, the fluorescent dyes rhodamine 123 (Rho-123) and 5(6)-carboxyfluorescein diacetate (CFDA) were used. Synovial sarcoma and leiomyosarcoma cells were incubated with 0.5 and 0.05 µM of Rho-123 respectively, or with 0.5 µM of CFDA dissolved in complete medium for 30 min at 37°C. After removing the extracellular free dye, the cells were incubated in dye-free media containing vehicle (DMSO), verapamil (10 µM), probenecid (250 µM), nilotinib (1–10 µM) or imatinib (1–10 µM). The efflux of Rho-123 and CFDA was analyzed at different time points (2 or 24 h) by flow cytometry (Beckman Coulter Epics XL flow cytometer).

### P-gp and MRP-1 immunohistochemistry in clinical samples

P-gp and MRP-1 expression was retrospectively analyzed in a subset of fifty two high-risk (grade III T2b N0 M0) localized STS tumors of limbs or trunk wall, which derived from a total of three hundred and twenty-eight patients enrolled in a randomized clinical trial conducted by Italian (ISG) and Spanish sarcoma groups (GEIS) focusing on adjuvant chemotherapy treatment [22]. These cases were enrolled prospectively in GEIS centers and each center received approval of its corresponding IRB. In the case of the Coordinating Center, the approval was given by the Ethical Committee of Clinical Investigation of the Balearic Islands in September 12th 2002. The main IP for Spain was Javier Martín Broto (medical oncologist). The Spanish Agency for Medicines and Health Products released its approval by May 12th, 2003. Written informed consent was obtained from all participants involved in this clinical trial. Three-micrometer sections of paraffin tissue blocks, fixed with 4% formaldehyde, were dewaxed in xylene, washed with PBS and pre-trated with citrate buffer (pH 6). Before staining the sections, endogenous peroxidase was blocked with 3% hydrogen peroxide. Immunohistochemical staining for P-gp or MRP-1 was performed incubating the sections with the monoclonal antibodies against P-gp (anti-P-gp dilution 1∶20) (SIG-38720 from Sigma, Covance, USA) or MRP-1 (QCRL-1 dilution 1∶100) (sc-18835 from Santa Cruz, USA) during 30 and 40 min, respectively. After incubation immunodetection was performed with EnVision FLEX (Dako, Glostrup, Denmark), employing diaminobenzidine chromogen (DAB) as substrate. Sections were counterstained with hematoxylin. Results were independently analysed by two pathologists who evaluated P-gp and MRP-1 expression qualitatively: positive (>10% positive cells) or negative. Tumor samples were available from all patients at the time of diagnosis so that only endogenous P-gp or MRP-1 expression was analyzed.

### Data analysis

The results are expressed as mean ± SEM. One-way analysis of variance (ANOVA) followed by Bonferroni's multiple comparison test was used for statistical evaluations. Differences were considered statistically significant at *P*<0.05. All calculations were done by GraphPad Software.

## Results

Growth inhibition by DXR, nilotinib and imatinib as single compounds was determined over a period of 72 h in synovial sarcoma SW982 and leiomyosarcoma SK-UT-1 cells. The average IC_50_ values for DXR and nilotinib in these cell lines were 1.30±0.07 µM and 0.13±0.09 µM, and 7.55±0.05 µM and 27.04±0.05 µM, respectively. At 10 µM nilotinib inhibited the growth of SK-UT-1 and SW982 cells by 33.5 and 59.6%, whereas imatinib showed only a mild effect on cell viability (inhibition of 12.1–19.1%) at the clinically achievable concentration of 10 µM [23] (Fig. 1D and 2B), with an IC_50_ of 26.48±0.02 µM and 89±0.02 µM, respectively.

We then investigated whether molecular targeted therapy (nilotinib and imatinib) might improve cell susceptibility to DXR, which is the standard drug in conventional chemotherapies for STS. To reveal the type of interaction between nilotinib and DXR concerning growth inhibition, an isobolographic analysis was performed and the combination index (CI) determined (as explained in Materials and Methods). Nilotinib (0.1–40 µM) and DXR (0.1, 0.3 and 0.5 µM) applied simultaneously for 72 h were highly synergistic in synovial sarcoma SW982 cells (with CI of 0.74, 0.49 and 0.58, respectively; Fig. 1A and B). Likewise, combination of nilotinib (2.5–10 µM) with DXR (0.1 µM) resulted in increased cell death, raising 2.0±0.35 and 4.5±0.79-fold the apoptotic effect of nilotinib and DXR, respectively (Fig. 1C). This synergism was also displayed by significant enhancements of cleaved caspase 3 and PARP, which were evaluated by western-blotting analysis (Fig. 1C, upper pannels), as well as enhancement of caspase 3 and 7 activities. Activated caspase 3/7 increased from 100±2.3% in vehicle-treated cells to 114.5±16.5% (n = 4; *P*>0.05) in DXR-treated cells, 235.3±12.2 (n = 4; *P*<0.001) in nilotinib (10 µM)-treated cells and 333.9±34.2% (n = 4; *P*<0.01 versus nilotinib- and *P*<0.001 versus DXR-treated cells,) in nilotinib plus DXR-treated cells. On the other hand, addition of nilotinib 24 hours before DXR exposure (0.05 µM) was the most effective combination sequence in leiomyosarcoma cells. In these cells nilotinib also synergized with DXR regarding apoptosis induction, although nilotinib did not induce apoptosis alone even after 96 h of treatment (Fig. 2A and C). Addition of nilotinib (2.5–10 µM) 24 h before DXR enhanced the apoptotic effect of a 72 h DXR treatment, increasing 1.8±0.21, 1.8±0.29 and 2.2±0.17-fold the level of DXR-induced cell death. Interestingly, further studies of apoptotic events also indicated the involvement of caspase 8, 3 and 7. Thus, in leiomyosarcoma cells combination therapy enhanced DXR-induced cleavage of caspase 8, 3 and PARP, which was evaluated by western-blotting analysis (Fig. 2C) as well as DXR-induced caspase 3 and 7 activities (activated caspase 3/7 increased from 100±0.7% in vehicle-treated cells to 247.5±15.0% (n = 4; *P*<0.001) upon treatment with DXR and 342.1±30.5% (n = 4; *P*<0.001 versus DXR-treated cells), 383.9±5.4% (n = 4; *P*<0.001 vs DXR-treated cells) and 412.9±20.7% (n = 4; *P*<0.001 versus DXR-treated cells) upon treatment with pre-nilotinib (2.5, 5 and 10 µM) plus DXR, respectively). In contrast, imatinib was not able to enhance the cell growth inhibition caused by DXR in any of the experimental settings (Fig. 1D and 2B).

Cell proliferation after withdrawal of compounds at the end of treatment was studied to investigate whether the synergism provoked by the combination of nilotinib with DXR was rapidly reversible or maintained during prolonged time. Leiomyosarcoma cells treated with nilotinib alone for 96 h showed increased cell number after drug withdrawal similar to vehicle-treated cells, whereas cells treated with DXR grew somewhat slower than control cells (Fig. 3). In contrast, combination of nilotinib (5–10 µM) with DXR significantly reduced by 46.9±3.9% and 70.3±5.8% cell number when compared to DXR treatment, even 12 days after the end of treatment (Fig. 3).

AKT and MAPK (ERK1/2 and p38 MAPK) are important signal transduction proteins implicated in growth factor-induced proliferation and survival of cancer cells in STS, especially in synovial sarcoma. SW982 cells showed low Akt, ERK1/2 and p38 MAPK phosphorylation after FBS starvation (lane 1, Fig. 4), but a high degree of activation when they were stimulated with 10% FBS for 30 minutes (lane 2, Fig. 4). Nilotinib significantly decreased FBS-induced phosphorylation of AKT, reaching a maximal inhibition at 10 µM of 43.2±9.0% when compared to vehicle-treated cells (Fig. 4). Furthermore, FBS-induced phosphorylation of ERK1 and p38 MAPK was fully blocked by nilotinib. Imatinib (10 µM) only slightly inhibited FBS-induced ERK-1 and p38 MAPK phosphorylation by 29.9±2.3% and 19.4±2.3%, respectively, and did not affect AKT activation when compared to vehicle-treated cells. Total AKT, ERK1/2 and p38 MAPK levels remained unchanged (Fig. 4).

To address the question whether the improved sensitivity of sarcoma cells to DXR induced by nilotinib might be associated with increased accumulation of DXR in these cells, intracellular DXR levels were evaluated in the presence or absence of nilotinib. Nilotinib applied simultaneously with DXR (1 µM) augmented DXR accumulation in SW982 cells after 24 h, raising its levels to 1.9±0.09- and 2.1±0.12-fold at a dose of 5 and 10 µM nilotinib, respectively (Fig. 5). Similarly, nilotinib pre-treatment also increased dose-dependently DXR accumulation in leiomyosarcoma cells by 1.4±0.02- and 1.6±0.18-fold, respectively (Fig. 5). In contrast, imatinib even at the highest dose did not significantly increase intracellular DXR in synovial sarcoma cells. Intracellular fluorescence intensity in imatinib- or nilotinib-treated cells was not different from vehicle-treated cells (data not shown). These results indicate that nilotinib, but not imatinib, enhances intracellular DXR accumulation.

The effect of nilotinib on the activity of the MDR-relevant efflux pumps P-gp and MRP-1 was then evaluated using the fluorescent dyes Rho-123 and 5-CFDA, which are known P-gp and MRP-1 substrates, respectively. In order to measure the efflux activity of P-gp in SK-UT-1 cells a lower concentration of Rho-123 (0.05 µM versus 0.5 µM) and a longer efflux time (24 h versus 2 h) than in SW982 cells had to be used because of the lower expression of P-gp protein in SK-UT-1 cells. Nilotinib (5–10 µM) increased the intracellular retention of Rho-123 by 2.4±0.30- and 1.6±0.08-fold in synovial sarcoma and leiomyosarcoma cells, respectively (Fig. 6), presenting higher efficacy than the P-gp inhibitor verapamil (10 µM) used as positive control. In a similar way, nilotinib also increased intracellular 5-CFDA accumulation by 11.8±0.45 and 11.1±0.33-fold in the above cell lines, being also more effective than the MRP-1 inhibitor probenecid (250 µM) used as positive control (Fig. 7). In contrast, the maximal dose of imatinib (10 µM) only slightly, but not significantly, increased intracellular Rho-123 and 5-CFDA by 1.2±0.06% and 1.2±0.07 fold in synovial sarcoma cells and by 1.3±0.07 and 1.5±0.13 fold in leiomyosarcoma cells, respectively (Fig. 6 and 7).

In addition, nilotinib (10 µM, 72 or 96 h) also down-regulated cellular expression of P-gp by 55.1±8.9% when compared to vehicle-treated cells (Fig. 8A). Importantly, incubation of synovial sarcoma cells with DXR (0.1 µM, 72 h) provoked a significant upregulation of P-gp levels (55.2±21.0%), which is generally considered as the central molecular mechanism of MDR in response to DXR treatment and which was fully blocked by nilotinib (Fig. 8A).

The fact that nilotinib eliminated FBS-induced p38 MAPK phosphorylation (Fig. 4) and that inhibition of this kinase reduces MDR in some human cancer cells by modulating P-gp expression [24], [25], suggested to analyze the effect of nilotinib and DXR on p38 MAPK phosphorylation. As shown in Fig. 8B, treatment of cells with DXR (0.1 µM, 72 h) increased p38 MAPK phosphorylation by 80.7±29.9% in synovial sarcoma cells when compared to vehicle-treated cells, whereas nilotinib treatment (10 µM, 72 h) strongly inhibited basal phosphorylation by 60.2±4.6%. When administered simultaneously nilotinib completely blocked DXR-induced activation of p38 MAPK (Fig. 8B).

Defining P-gp and MRP-1 as additional therapeutic targets of nilotinib to enhance antitumoral efficacy of DXR in STS would only be, if these proteins were expressed in the clinical context. Thus, endogenous P-gp and MRP-1 expression was qualitatively and retrospectively evaluated by immunochemistry in a subset of 52 high-risk advanced STS tumors of limbs or trunk wall obtained from 328 patients enrolled in an international randomized trial [22], and positive immunoreactivity for P-gp was observed in 28 cases (53.8) and for MRP-1 in 8 cases (15.4%) (Table 1). Coexpression of both MDR-related proteins was found in 6 (11.5%) tumor samples. From the 11 and 4 cases of synovial sarcoma and leiomyosarcoma, 8 (72.7%) and 4 (100%) tumor samples were evaluated as P-gp-positive sarcomas and 3 (27.27%) and 1 (25%) as MRP-1-positive ones, respectively (Table 1).

## Discussion

TK inhibitors are small molecules, whose mechanism of action is ATP-binding site blockade of specific TKs and TK receptors, thereby suppressing downstream pathways linked to cell proliferation. In this context, the TK inhibitors imatinib and nilotinib are able to inhibit the activity of the TK BCR-ABL as well as the activity of the TK receptors KIT and PDGF, being nilotinib more potent and selective against BCR-ABL1 [12]. In contrast to classical chemotherapeutics both compounds were developed for a defined molecular target and imatinib shows clinically impressive results in patients with CML or advanced GIST, in which overexpression or gain of function of one of the above mentioned kinases leads to malignant cell growth [6], [7]. More importantly, nilotinib maintains antitumoral activity against the majority of imatinib-resistant mutants, and has a better side-effect profile than imatinib [14], [26], [27]. Although some gene array and immunohistochemistry studies in tumor tissues of patients with STS have revealed that certain TK receptors (e.g., KIT, PDGFβ) are overexpressed [28], [29], at present it is still unclear whether TK inhibitors as single agents or combined with conventional chemotherapy are effective on cellular models of STS others than GIST [30] and Ewing tumor cells [31].

Our study demonstrates that the novel TK inhibitor nilotinib exhibits higher antiproliferative activity than imatinib in two human STS cell lines, whereby synovial sarcoma SW982 cells appear significantly more sensitive than leiomyosarcoma SK-UT-1 cells. The potency of nilotinib in these STS cells cannot be explained by the currently declared mechanism of action of this drug, namely inhibition of the catalytic activity of BCR-ABL1, KIT and PDGFR-α/β, because the concentration of nilotinib required to inhibit these TKs in cellular models is in the nanomolar range [32], [33]. This idea is strengthened by the fact that imatinib, at clinically achievable plasma concentrations of 5–10 µM [23] decreased cell viability only between 12–19% (Fig. 1D and Fig. 2B). Even if indirect, our results suggest that novel mechanisms may be involved, such as inhibition of other tyrosine or non-tyrosine kinases. In this context, suppression of proliferation by imatinib and nilotinib correlated with their capability to down-regulate AKT and mitogen-activated protein kinases (MAPKs) (Fig. 4). MAPK cascades, especially ERK and p38 MAPK, promote growth factor-mediated proliferation, survival and resistance to apoptosis in STS, including synovial sarcoma. Consequently, inhibition of MEK/ERK and p38 MAPK is able to suppress proliferation in several synovial sarcoma cell lines, including SW982 cells [34], [35]. In line with the observed effect on cell growth, nilotinib but not imatinib fully blocked basal and FBS-induced ERK1 and p38 MAPK activation in SW982 cells. Although this effect might be mediated through blockade of upstream TK receptor signaling, another plausible explanation would be a direct inhibitory interaction between nilotinib and ATP-dependent p38 MAPK. In support of this hypothesis, kinase binding assays have demonstrated substantial binding affinity of nilotinib to p38 MAPK, which translated into potent inhibition of the catalytic activity of p38 MAPK in enzymatic assays [33]. Furthermore, nilotinib is more lipophilic and relatively less protonated at physiologic pH than imatinib [27], [36], thus, facilitating its plasma membrane passage and accessibility of intracellular interaction partners. For these reasons, it is conceivable that p38 MAPK may be a further important molecular target of nilotinib, in particular with respect to synovial sarcoma cells. Besides contributing to aberrant cell proliferation, p38 MAPK activation also participates in the development of MDR by modulating the activity and expression of the P-gp protein [24], [25], [37]. P-gp and MRP-1 proteins belong to a large family of ATP-dependent transporters located in the plasma membrane, which extrude a large variety of compounds, including chemotherapeutic agents, out of the cells, thereby decreasing their intracellular concentrations and consequently reducing their pharmacological efficacy. Clinical chemotherapy is often counteracted by MDR in which overexpression of these proteins in tumor cells is frequently the prevailing antitumoral resistance mechanism [38]. Accordingly, in our study synovial sarcoma SW982 cells also quickly developed this typical feature of MDR, i.e. the induction of P-gp expression in response to DXR treatment (72 h) (Fig. 8A). In this experimental set nilotinib prevented DXR-induced MDR through inhibition of P-gp and MRP-1 activity (Fig. 6 and 7) and cellular up-regulation of P-gp, which was to be expected because of the detected blockade of p38 MAPK activation by nilotinib (Fig. 8B). Consequently, nilotinib increased the accumulation of DXR inside STS cells (Fig. 5) and enhanced its antiproliferative and apoptotic effects (Fig. 1 and 2), which was further confirmed by an enhancement of DXR-induced cleavage of several caspases (caspase 8, 3 and 7) and fragmentation of PARP. Importantly, STS cells relatively quickly regained their neoplastic rate of proliferation after nilotinib or DXR treatment, whereas the sensitization effect of STS cells to DXR induced by nilotinib was detectable during long time, since cell number was found strongly reduced even 12 days after withdrawal of both compounds (Fig. 3). This is in line with the observation that combination therapy leads to a higher intracellular level of DXR in tumor cells, which probably requires longer time to be removed from them. Increased sensitivity to DXR was only detected in STS cells expressing P-gp and MRP-1, since nilotinib did not alter intracellular DXR concentration in human fetal lung fibroblasts (MRC-5) (intracellular DXR fluorescence 1.3-fold in the presence of 5 µM nilotinib compared to DXR alone). This observation is consistent with results obtained by Tiwari and colleagues who just recently reported that nilotinib at 2.5–5 µM reverted P-gp- and ABCG2-mediated resistance by inhibiting their activity, without affecting their expression, in human embryonic kidney cells transfected with these MDR-related proteins [16]. The lower antiproliferative effect of imatinib and its inability to sensitize STS cells to DXR may be partially explained by the facts that i) intracellular uptake of imatinib, but not nilotinib, depends on expression of organic cationic transporter-1 (OCT-1) influx protein [36], [39], ii) imatinib is a substrate for P-gp provoking a reduction of its intracellular levels and, iii) in contrast to nilotinib, imatinib did not show any inhibitory effect on P-gp activity in P-gp expressing cells at the concentrations examined [40].

Different chemotherapeutics agents have been tested in STS [41], but this class of solid tumors, particularly synovial sarcoma, is difficult to treat by chemotherapy. Up to date DXR still remains the most effective agent and is considered the systemic standard treatment in metastatic setting throughout the last twenty years [1]. Nevertheless, neither therapies with DXR alone nor in combination with ifosfamide have achieved significant improvements in terms of overall survival [42]. In addition to its high cytotoxicity, some tumors become resistant to this chemotherapeutic due to development of MDR, mainly characterized by overexpression of P-gp and MRP-1 [3], to which has been attributed the failure of cancer therapy in over 90% of patients. In this context, in a subset of 52 high-risk locally advanced STS tumor patients derived from a large international study with 328 participants [22] 54% and 15% were positive for P-gp and MRP-1 expression, respectively, with high incidence of P-gp in synovial sarcoma (73%). Together our results suggest that nilotinib could be a readily available therapeutic alternative, as single compound or combined with DXR, for treatment of this type of solid tumors in general but above all for synovial sarcoma. Although nilotinib at clinically achievable plasma concentrations of 5 and 10 µM had only moderate antitumoral effects, combination of nilotinib with DXR was highly synergistic in both STS cell types. Because of the wide substrate profile of P-gp in relation to chemotherapeutic agents [43], it is tempting to speculate that nilotinib could also enhance the antiproliferative effects of other antitumoral agents affected by MDR, such as paclitaxel or vinblastine. Noteworthy, an amplification of the dose-limiting toxic effects of classical antitumoral agents through co-treatment with TK inhibitors should not have to be expected. In fact, the clinical viability of such a combination strategy regarding possible adverse side effects has already been successfully tested using imatinib plus DXR in patients with imatinib-resistant GIST [44].

In summary, the present study reveals that the TK inhibitor nilotinib not only exhibits considerable antiproliferative effects on STS cells, but also, and in contrast to imatinib, sensitizes them to DXR by blocking the activity of the MDR-related proteins MRP-1 and P-gp as well as the DXR-induced expression of P-gp in synovial sarcoma, the latter most probably through inhibition of p38 MAPK. These results are of direct clinical relevance as they provide molecular evidence for the assessment of a novel chemotherapeutical strategy using nilotinib and DXR as treatment combination especially in synovial sarcoma. This treatment approach could ameliorate treatment outcome of patients without increasing drug toxicity and the official regulatory process to obtain approval for a clinical study has recently been initiated.
